# Study on stress variation of advance fiberglass anchor bolts during tunnel excavation process

**DOI:** 10.1038/s41598-023-31000-4

**Published:** 2023-03-13

**Authors:** Jimeng Feng, Yumei Tan, Shiyu Yao, Hui Jiang, Junru Zhang, Hongtao Li

**Affiliations:** 1 School of Civil Engineering, Southwest Jiaotong University, Chengdu, 610031 Sichuan China; 2 Key Laboratory of Transportation Tunnel Engineering, Ministry of Education, Southwest Jiaotong University, Chengdu, 610031 Sichuan China; 3 Department of Civil Engineering, University of Birmingham, Birmingham, B15 2TT UK

**Keywords:** Civil engineering, Mechanical properties

## Abstract

One of the main causes for excessive deformation within a tunnel is due to the instability of the soil or soft rock ahead of the excavation face. Fiberglass bolts have been shown to be a useful advance reinforcement method for the excavation face. In this paper, an improved ADECO-RS (Analysis of controlled deformation in rock and soils) method had been proposed for soft rock mountain tunnels, in terms of the partial (mainly the upper bench) excavation face reinforcement and also for the bench excavation method. Strain gauges were used to test the micro-strain in the fiberglass bolt to investigate how the axial force of the fiberglass bolt varied during the tunnel excavation. In addition, combined with the field tunnel deformation monitoring data, the relationship between the reinforcement parameters of the fiberglass bolts and the tunnel construction phase were discussed. The research results show that: (1) The stress state of the anchor rod is related to the reinforcement length of the anchor rod; (2) Excavation within the lap area of the fiberglass bolt leads to an increase in the axial force of the bolt, while excavation outside the lap area of the fiberglass bolt has no effect on the anchor; (3) Reducing the reinforcement area of rock mass will affect the stability of the excavation. To ensure the stability of the excavation face, the initial support construction loop should be completed as soon as possible; (4) In a future project with similar conditions, the recommended lap length of the fiberglass bolt could be 3 m utilizing the fiberglass bolt grouting face reinforcement method.

## Introduction

With the rapid development of infrastructure in China, a large number of new train lines and associated tunnels are being planned or constructed in the mountainous regions. Especially in southwestern China, there are many mountainous areas with complex geological conditions. Large deformation of the tunnel is one of the most frequent problems facing engineers during tunnel excavation in southwest of China due to the weak surrounding rock. A large number of researchers have put a lot of effort into understanding and reducing this problem all over the world^1,2^ Out of all the methods dealing with this engineering problem, the construction method ADECO-RS (Analysis of controlled deformation in rock and soils) proposed by Italian scholar Lunardi has achieved good results in controlling large tunnel deformations^3,4^. The research results of Tonon^5^ have shown that this method has been successfully applied to tunnels with super-large Sects. (260m^2^). Based on the concept of this method, the stability of the tunnel is directly affected by the extrusion deformation of the rock or soil directly ahead of the excavation face. Hence, strengthening the rock or soil ahead of the excavation face can not only prevent the collapse of the tunnel face but also enable full-face excavation, which helps to complete the full circumferential initial support construction as early as possible.

In terms of reinforcement methods for stabilizing the soil to be excavated ahead of the excavation face, the use of fiberglass bolts is one of the most commonly adopted methods^6^. As the parameters of the fiberglass bolt (i.e., diameter, spacing, length, lap length) are directly related to the size of the excavated face and the strength of the rock mass, many researchers have focused their research on the instability mechanisms and reinforcement effects of the excavation face after using fiberglass bolts to guide the choice of bolt parameters. Meanwhile, extensive studies have revealed that improving the strength of the weak surrounding rock by reinforcing the excavated face is an important method to improve the excavated face's stability^7–12^. Perazzelli^13^ analyzed the stress distribution relationship around a bolt to understand the working mechanism of the bolt. Li^14^ used numerical simulation methods to analyze the influence of the fiberglass bolt parameters on the tunnel stability. The results showed that the core soil in the excavation face without bolts started to yield when the surrounding rock pressure reduced to about 60% of the initial horizontal earth pressure. Normally, the effect of reducing the face displacement tends to disappear when the length of the bolt exceeds 8 m, suggesting a critical length (Lcr) of 0.6H (H = height of tunnel). Alessandra^15^ analyzed the stability and deformation characteristics of the excavation face after bolt reinforcement, and suggested that the limit state of surface subsidence should be considered when choosing the bolt parameters during the urban excavation in soft rock.

Due to the difficulty in testing the internal force and displacement of bolts in the field, plentiful researchers have used numerical simulation methods to understand the stresses in the bolt^16–18^. Tan^19^ analyzed the extrusion deformation of the excavated face reinforced using fiberglass bolts by monitoring ground displacements in the Boar Mountain tunnel in Ningbo, China. The results showed that the core soil ahead of the excavation face within 6 m was significantly influenced by the excavation, so a lap length of 6 m for the bolts was recommended. In addition, it was found large deformations was occurring, especially after 4 h of the excavation, so reducing the time to construct the initial support to meet the 4 h has been suggested.

Pre-reinforced full section excavation based on the ADECO-RS method has been fruitfully investigated by many researchers. However, based on the current state of construction technology in China, partial (mainly the upper step) excavation face advanced reinforcement + step excavation method is a feasible combination to effectively control the deformation of the surrounding rock. In contrast to the full-face excavation used in the ADECO-RS method, the stress distribution and excavation stress evolution in face reinforcement for partial face construction present some differences. For the partial excavation face advanced reinforcement + step excavation method, the effect of its pre-reinforcement measures on the soil in front of the excavation face is still in the initial stage.

Therefore, this paper proposed a new excavation method (the improved ADECO-RS method for soft rock mountain tunnels) for the partial excavation face advance reinforcement + step excavation method. Considering the differences in numerical calculations with the field situation, field data should better reflect the realities of tunnel excavation. Compared with the numerical method, the fiberglass bolt in the field cannot be constructed simultaneously. The stress balance was gradually achieved with the redistribution of surrounding rock stress. So the research presented in this paper chose to use field monitoring data to analyze the ADECO-RS approach. Even though the stresses in the bolt at the start were different, the bolts were considered under the same force, which means the relationship for the bolts was the same. This paper applied the proposed method to the construction of a large deformation tunnel section and studied the variation of stress in the excavation face reinforcement bolts using the micro-strain field test of the fiberglass bolt. In addition, the relationship between the bolt axial force during the tunnel excavation relative to the bolt parameters and the deformation was investigated.

### Tunnel project background

**Figure 1:**
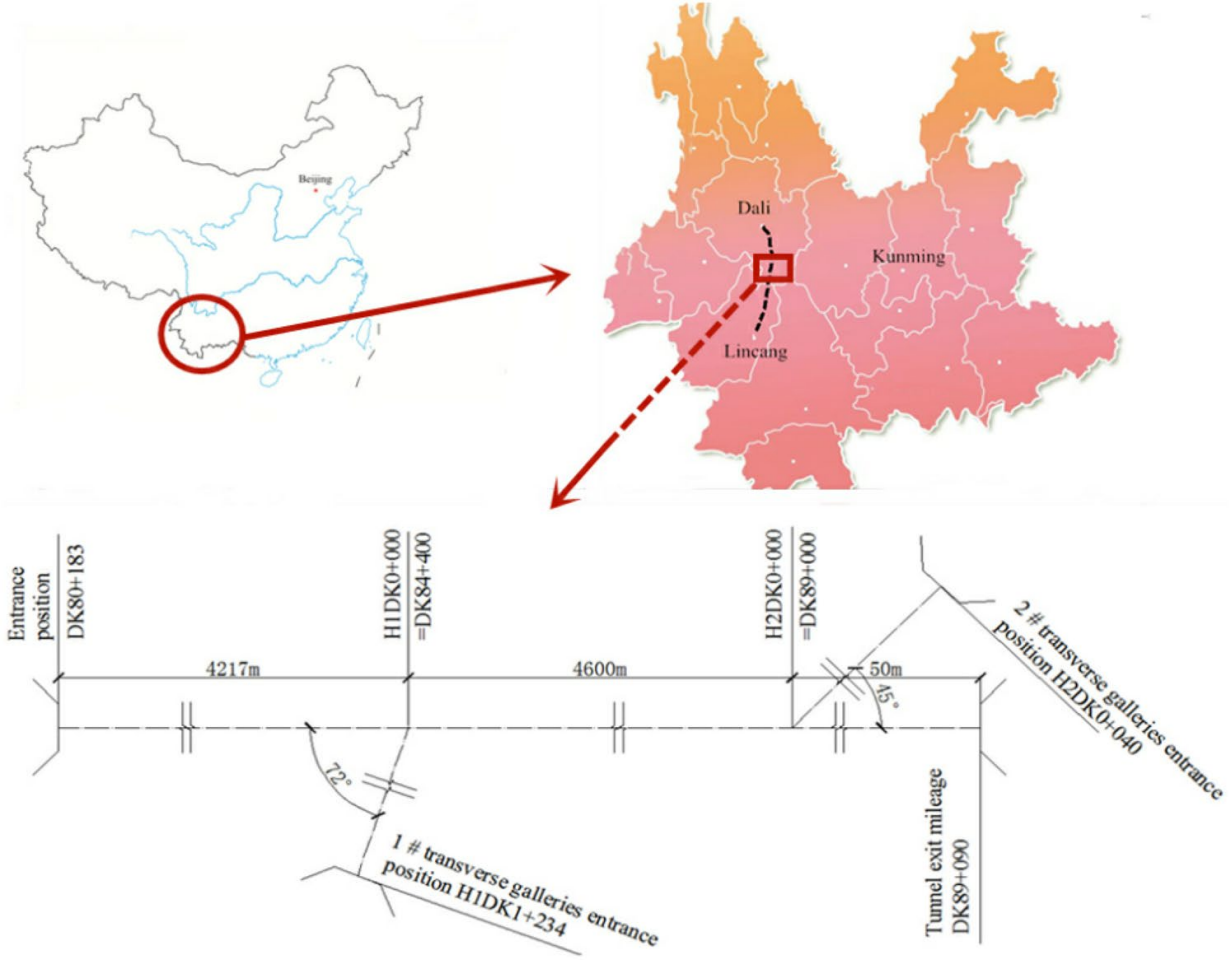
Location and layout of the Xingzishan tunnel.

**Figure 2:**
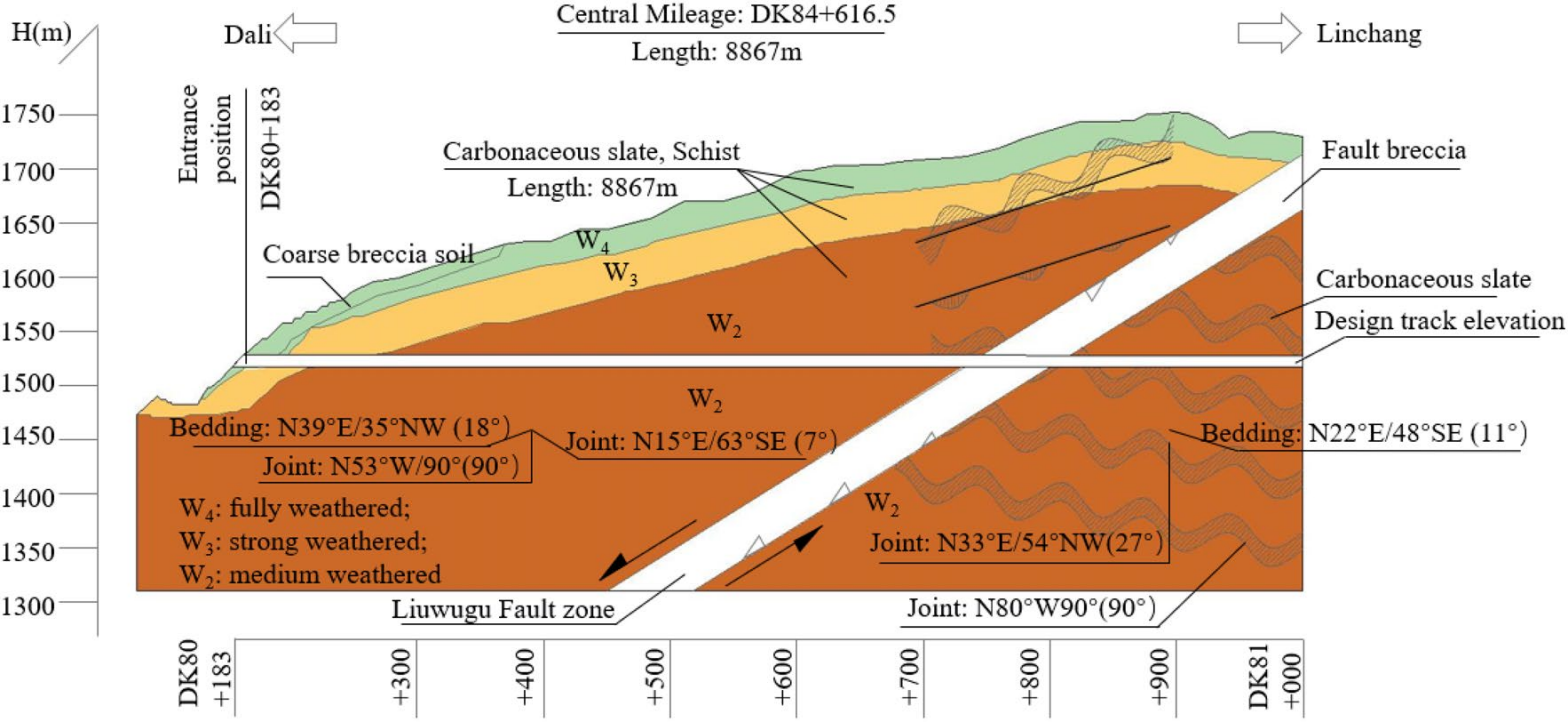
Longitudinal geological section through the tunnel from the tunnel portal.

**Figure 3:**
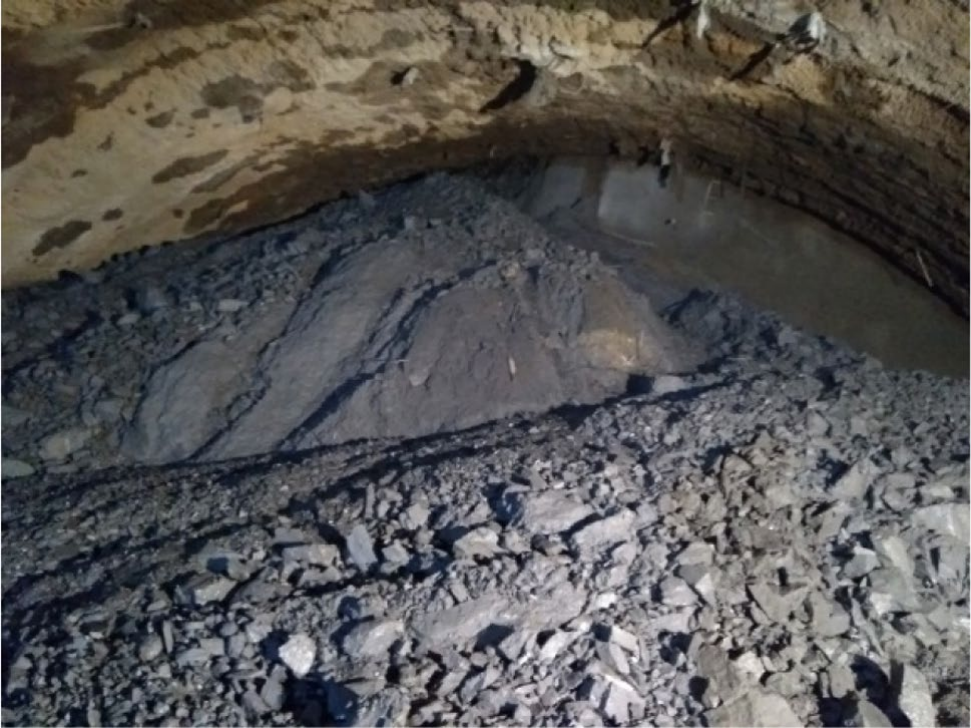
An example of the surrounding rock condition after excavation.

The Xingzishan tunnel is one of the important infrastructures along the Dalin railway, and the location is shown in Fig. 1. The total length of the tunnel is 8867 m, and the size of the excavation face is approximately 143.64 m^2^. In addition, the height and width of the tunnel are approximately 11.24 m and 14.47 m, respectively. The longitudinal geological section through the tunnel centreline is shown in Fig. 2. The tunnel contains a double track 819 m long. The large deformation problems mainly occurred close to the portal section of the tunnel. Based on the geological exploration, the strata mainly consisted of: mudstone and sandstone in the lower member of the Jurassic Huahuazuo Group (J_2_^h1^); Mudstone and sandy mudstone mixed with sandstone in the lower member (J_1_^y^) of the lower Yangjiang Group; The fifth member (∈ wl5) of the Wuliang Mountain Group of the Cambrian System is dolomitic limestone, limestone, carbonaceous slate, sandy slate, phyllite mixed with schist and metamorphic sandstone, and the fourth member (∈ wl4) is carbonaceous slate, calcareous slate, quartz schist and mica schist. The surrounding rock around the tunnel entrance is carbonaceous slate. As joints and fractures are highly developed in the strata, as shown in Fig. 3, field core samples could not be obtained.

**Figure 4:**
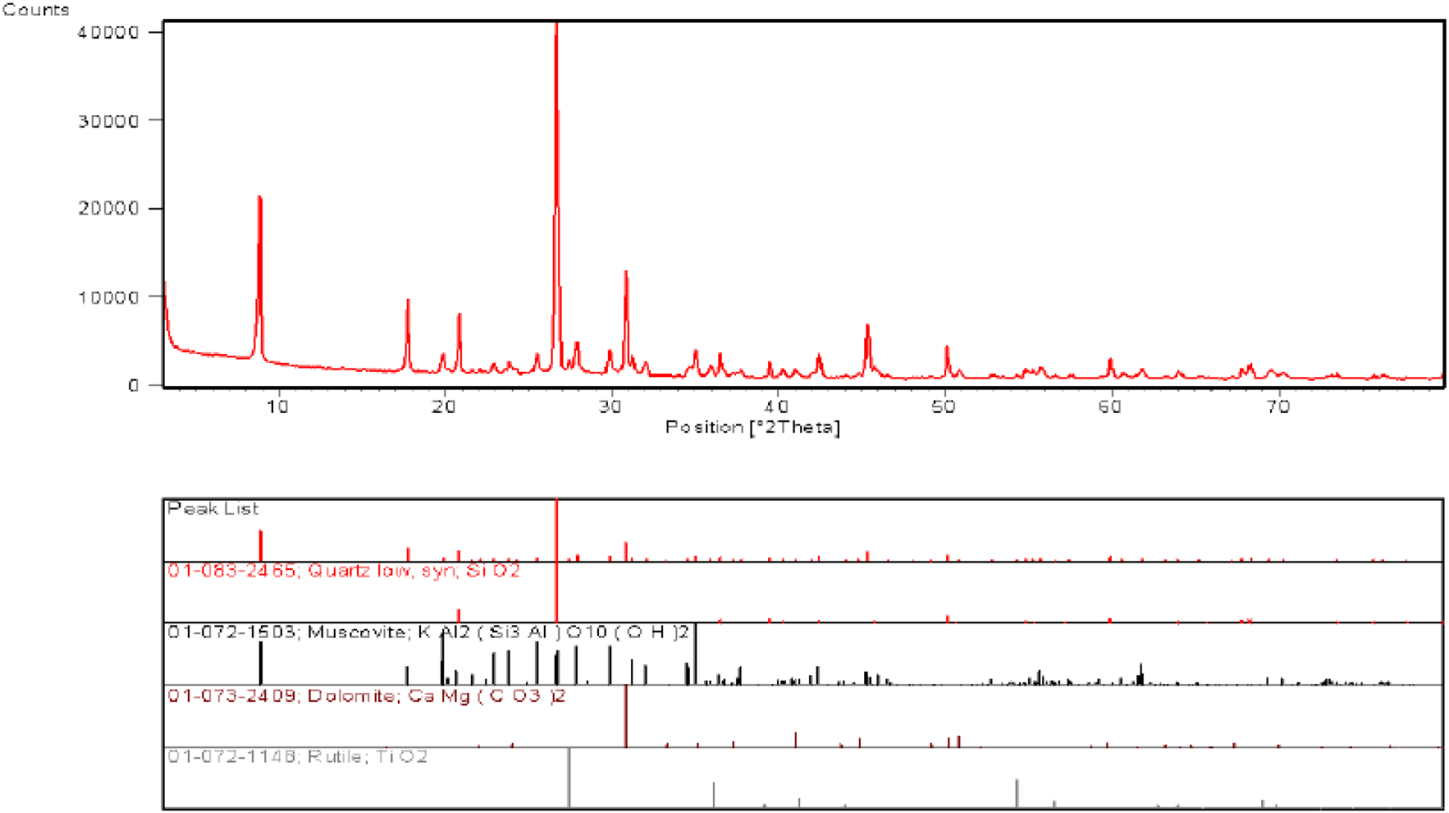
DK80 + 432 XRD sampling test results of left side excavation face.

Based on XRD tests of the samples as shown in Fig. 4, the mineral composition and content of the rock are shown in Table 1.

**Table 1:** Composition and content of the rock minerals.

Sampling location	Mineral composition	Mineral content(%)
DK80 + 432 Tunnel face on the left side	quartz	33
	muscovite	51
	dolomite	33
	rutile	4
DK80 + 432 Tunnel face on the right side	quartz	31
	muscovite	39
	dolomite	31
	clinochlore	22
	rutile	3

The carbonaceous slate rock mass is mainly composed of schistose extreme cleavage silicate minerals such as muscovite and clinochlore, accounting for 50% ~ 60% of the total content. There is also a classic mineral quartz (the main component of the quartz is SiO_2_), with a relative content of more than 30%. There is also a small amount of rutile (mainly TiO_2_) with a content of 3% ~ 4%. There is very little montmorillonite, illite, kaolinite, and other clay minerals in the rock samples, which means the rock mass surrounding the tunnel had a low swelling potential.

**Figure 5:**
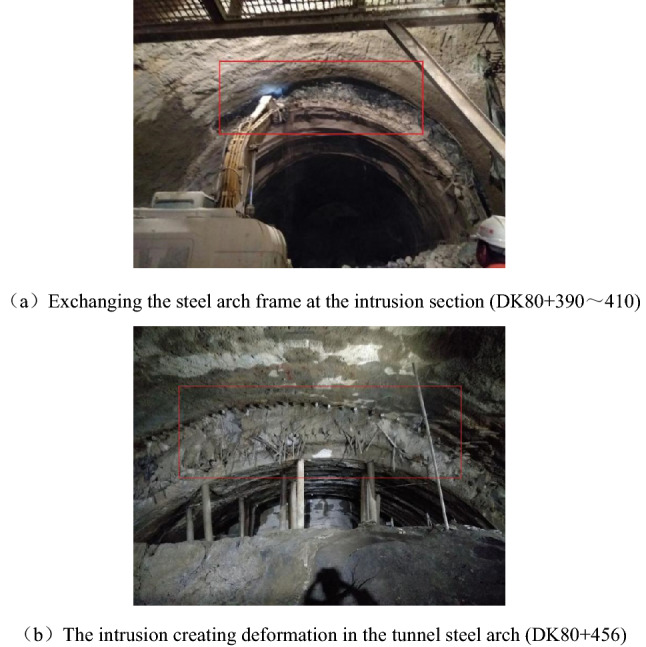
The intrusion of the surrounding rock after the construction of the initial support.

After the excavation of the portal section of the tunnel, there was severe deformation, and the settlement of the steel arches of the tunnel could not stabilize. The large deformation of the tunnel happened after the construction of the initial support was installed, and attempts to strengthen the initial support did not work very well with concerning controlling the deformation, shown as in Table2. For example, constructors used a double-layer I25b steel arch frame in DK80 + 446 ~ DK80 + 458 to control the deformation, but it failed, as shown in Fig. 5.

**Table 2:** Parameters for the tunnel support systems.

Segment	Initial support	Advanced support	Bolt
DK80 + 370 ~ + 410	Steel arch frame: Full ring I22b type, spacing 0.8 m(DK + 350 ~ + 370) or spacing 0.6 m(DK + 370 ~ + 390; Steel mesh: ф8/20 cm × 20 cm	Small pipe: ф42, the longitudinal spacing is 2.4 m, Lap 1.6 m, length 4 m, circumferential spacing 0.4 m, 42 pipes per ring	Arch: Ф 25 combined hollow anchor rod, side wall: Ф 22 mortar anchor rod, 4 m long, spacing of 1.2 m × 1 m (circumferential direction × longitudinal direction)
DK80 + 410 ~ + 446	Steel arch frame: Full ring I25b, spacing 0.6 m; I18 I-steel as longitudinal connector, circumferential spacing 1 m; Locking anchor pipe: 4 Φ42 at steel frame arch foot; Two 6-m-long φ 89 anchor pipes shall be added to the left and right arch feet of the upper step and grouted respectively; Steel mesh: Φ8/20 cm × 20 cm	Small pipe: Double Φ42, 4 m long, longitudinal spacing 2.4 m, circumferential spacing 0.3 m, 63 × 2 pipes per ring	Arch: Ф 25 combined hollow anchor rod, side wall :Ф 22 mortar anchor rod, 4 m long, spacing of 0.8 m × 1.2 m (circumferential direction × longitudinal direction)

**Figure 6:**
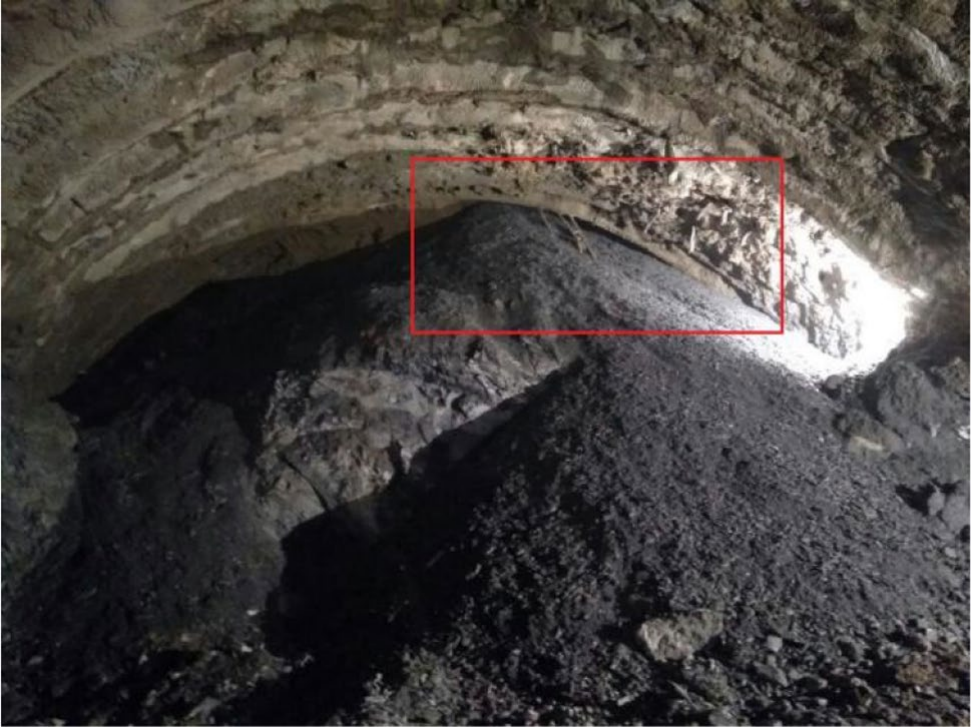
Collapse of the excavated face (DK80 + 407).

During the construction process, the collapse of the excavated face occurred because of groundwater softening, as shown in Fig. 6.

**Figure 7:**
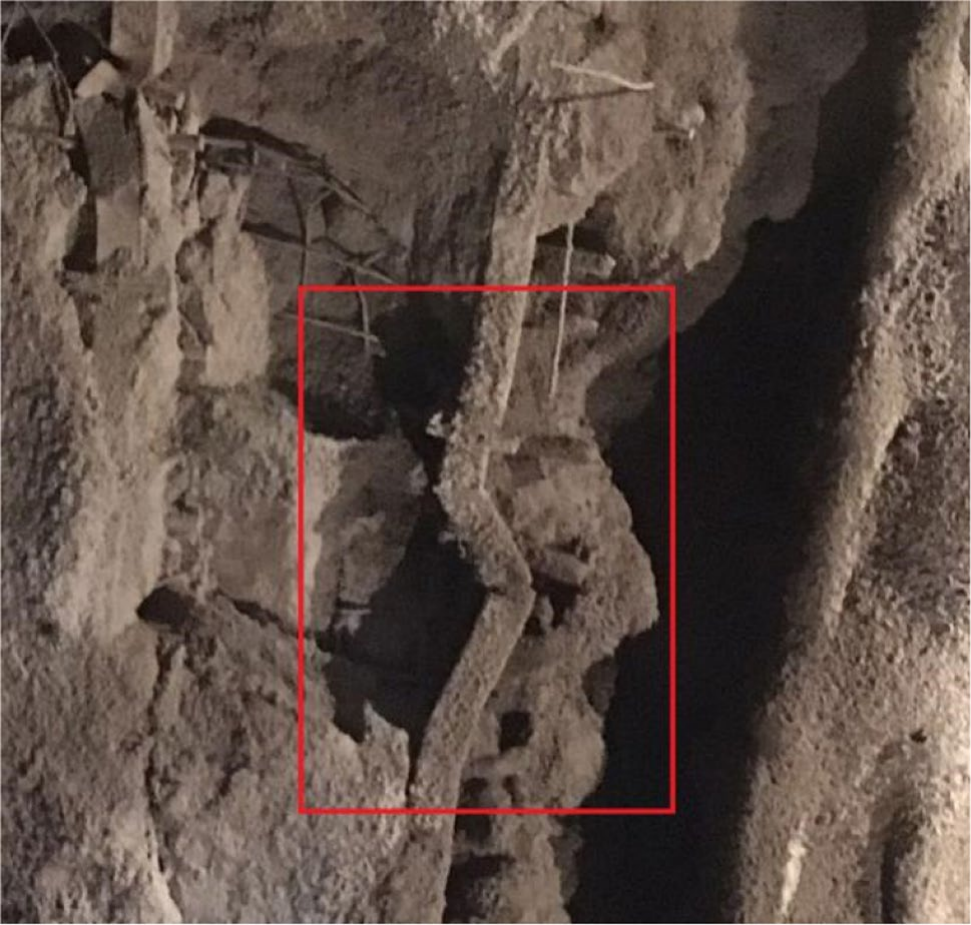
The failure of the steel arch frame at DK80 + 427.

The initial support (i.e., sprayed concrete) fell off, and there was deformation and twisting in the steel arch frame, as shown in Fig. 7.

**Figure 8:**
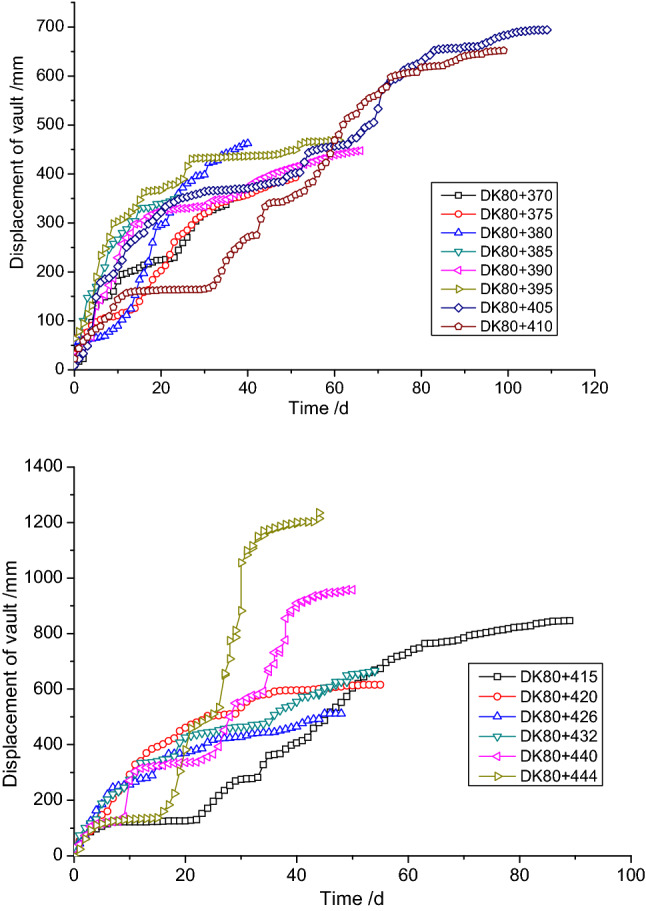
Monitoring data for the settlement of the vault at some large deformation sections in the tunnel.

Figure 8 shows the large deformations that occurred at some locations along the tunnel. Normally, the settlement of the steel arches didn’t become stable until 60 days after installation. The maximum deformation of the steel arch in DK80 + 444 reached 1.2 m, which far exceeds the deformation limits of these arches.

According to the field observations and the monitoring data, the deformation of the tunnel could not be well controlled simply by strengthening the supporting structures. In addition, the unstable excavated face of the tunnel was a significant factor affecting the deformation of the tunnel. With respect to the construction phase, the more supporting structures that needed to be installed, the greater construction time, meaning it would take longer to complete circumference of the initial support. Hence, the large proportion of the deformation that happened during the initial construction time could not be effectively controlled.

## Advance reinforcement of the excavation face to control deformation

Compared to the TBM (Tunnel Boring Machine) method, which is widely used in the construction of railways in China, the ADECO-RS method uses explosives to disturb the surrounding rock more than the TBM method. At the same time, the tunnel abandoned soil transport work of the ADECO-RS method increases the amount of labor. However, the ADECO-RS method considers the deformation of the surrounding rock in front of the excavation face and adopts the form of advanced support to control the deformation of the core soil in front of the excavation face. Therefore, the construction method has relatively low requirements on geological conditions and is more suitable for the construction of deeply buried tunnels with poor rock quality and large sections. It has achieved good results in the deformation control of the surrounding rock of roadway with large deformation.

Although China currently has a large tunnel construction program, there are few engineering cases of advanced reinforcement being used for full cross-section face excavation in large-section tunnels. One of the main reasons is that the construction techniques required for grouting the reinforcement for a full-section face of large cross-section tunnels have not been available, nor have the trained personnel to operate it. In addition, the higher cost and longer construction time are other significant reasons that this technology has not been used very often. It should be noted that based on evidence from previous studies, the use of partial advance support of the excavation face has been shown to control well the excavation face stability problems^20^.

**Figure 9:**
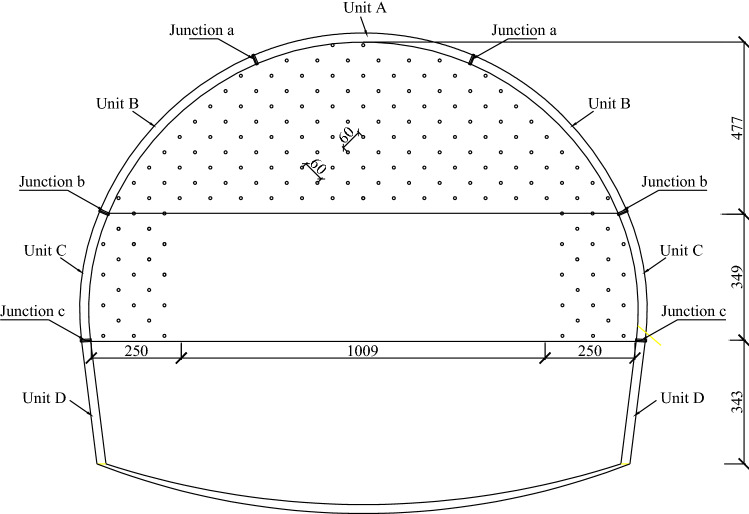
Arrangement of the fiberglass bolts within the excavated face.

**Figure 10:**
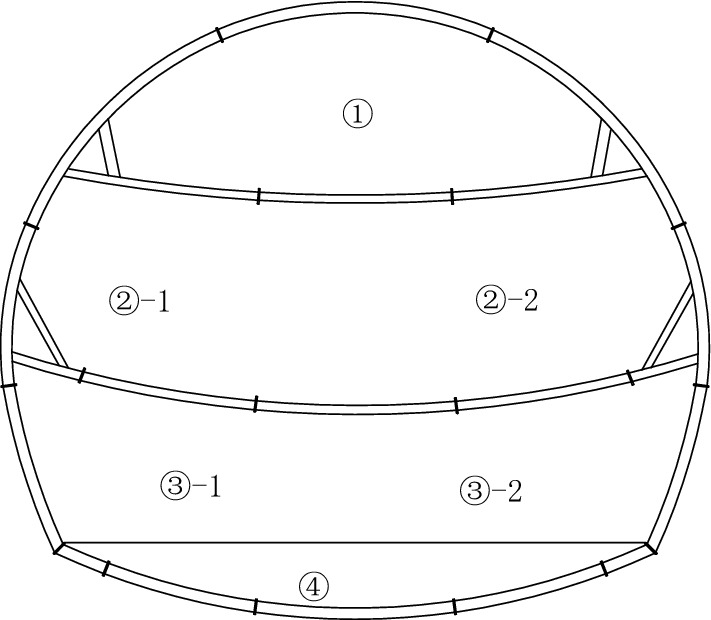
The three steps excavation method with temporary inverted arches.

Fiberglass bolts have the advantages of high tensile strength, low shear strength, fatigue resistance, non-conductivity, and corrosion resistance. Their low shear strength makes them susceptible to cutting during the tunnel boring process. Under the condition that the effect of improving the initial support parameters was not good for the large deformation sections of the Xingzishan tunnel, advanced grouting of the excavation face with fiberglass bolt and the temporary inverted arch. The design parameters for the advanced grouting with fiberglass bolt were: diameter of 25 × 7 mm; the length was 12 m; the spacing was on a grid of 0.6 m × 0.6 m and the construction section was once every 8 m, which meant that the overlap length of the bolts was 4 m, as shown in Fig. 9. The parameters of the fiberglass bolts are shown in Table 3. The excavation method was changed from the three steps excavation method with a reserved soil core to a three steps excavation method with temporarily inverted arches, as shown in Fig. 10. The construction steps used were as follows: initially excavate①, and then constructed the initial support and temporary inverted arch; excavate②, ③, ④as ①. Finishing④ means that the completed tunnel lining ring for the initial support is in place.

**Table 3:** Parameters for the fiberglass bolts.

Diameter/mm	Weight/g/m	Density/g/cm^3^	Elastic modulus /GPa	Ultimate tensile strength /MPa	Shear strength /MPa
25	680	1.9–2.1	≥ 40	≥ 750	≥ 110

**Figure 11:**
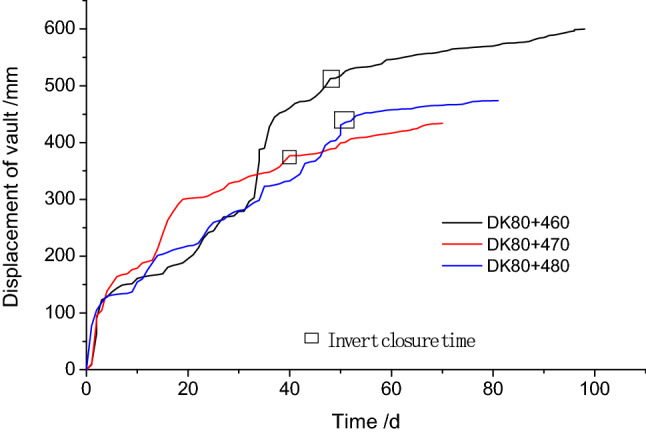
Displacement of the vault at three chainages for the three steps excavation method with temporary inverted arches.

As shown in Fig. 11, the displacement curve of the vault at selected chainages shows a greatly reduced overall deformation with time. Except the deformation of section DK80 + 460 near the section without taking advanced reinforcement measures have reached at 60 cm, the other sections was less than 50 cm. Once the initial support ring was completed with the inverted arch at the invert, the formation of the vault was stabilized. Data comparing the two methods is shown in Table 4. It can be seen from the overall deformations that the settlement of the vault and the convergence of upper and middle benches were greatly reduced, creating a maximum 45.52% reduction, which means the deformation of the surrounding rock was effectively controlled.

**Table 4:** Comparison of the surrounding rock convergence between the two construction methods (all values in mm).

	Vault	Upper left side	Upper right side	Middle left side	Middle right side	Lower left side	Lower right side
DK80 + 432	666.2	394.1	290.8	364.1	307	126	116
DK80 + 440	947.9	452.3	268.8	195.1	206.6	24.2	31.9
Average deformation	807.05	423.2	279.8	279.6	256.8	75.1	73.95
DK80 + 470	423.2	245.8	264.4	219.4	239	129.8	93.3
DK80 + 475	427.8	192.9	238.7	172.6	178.8	105.3	122.3
DK80 + 480	468	255.4	266.7	170.2	207	45.5	63.5
Average deformation	439.7	231.4	256.6	187.4	208.3	93.5	93.0
Deformation reduction percentage	45.52%	45.33%	8.29%	32.98%	18.90%	-24.55%	-25.81%

**Figure 12:**
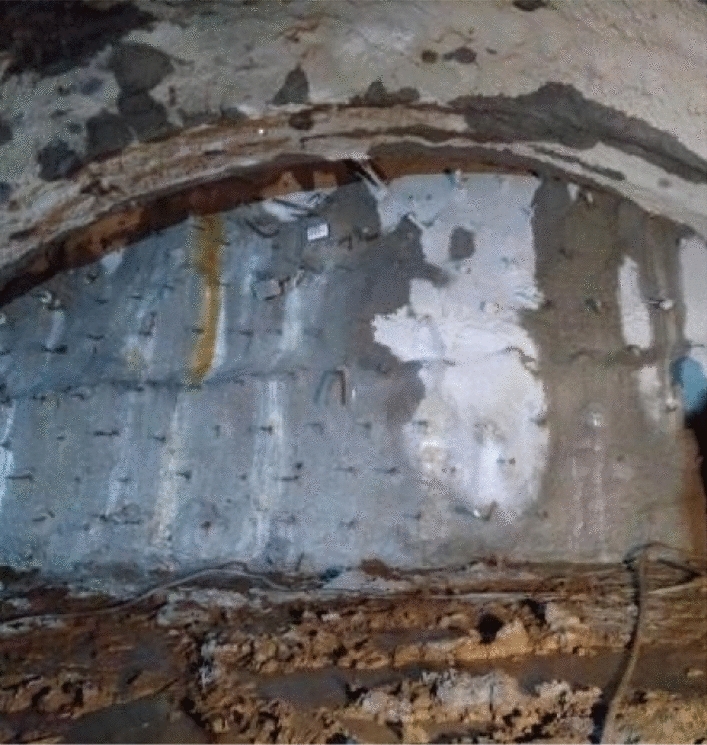
Example of the stabilized face.

As the excavation face of the lower step was not reinforced by grouting, its convergence was slightly increased compared with the data from the previous construction method, but this only have a small influence on the overall structure. The deformation and especially the face stability were effectively controlled using the revised construction method, as shown in Fig. 12.

**Figure 13:**
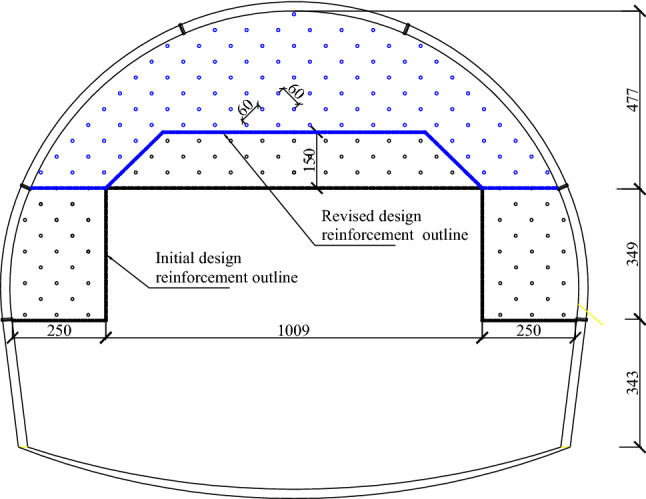
The reinforced face area before and after adjustment.

During the construction, some problems were encountered with the construction of the 12 m long fiberglass bolts, which resulted in a poor reinforcing effect. The installation of the 12 m bolt lead to long construction times for this process, and had knock-on effects for the whole tunnel construction as no other face excavation could take place during the bolt installations. Therefore, to improve construction times and considering that the deformation has been effectively controlled by the fiberglass bolts, it was proposed to adjust the length of the bolts to 9 m, with lap lengths of 3 m. The spacing of the bolts remained the same, but the reinforced area was reduced, as shown in Fig. 13. However, an investigation was required to ensure that the reduction in reinforcement length and lap length would have any influence on the stability control of the face and this is discussed in the next session.

## Testing method for determining the internal force in the fiberglass bolts

Grouted reinforcement with fiberglass bolts is a common method for advance reinforcement of the excavation face, and the stress state in the bolt is an important indicator of the reinforcement effectiveness. Due to the construction operations, the bolts are generally destroyed during the excavation process. Therefore, it is extremely difficult to monitor the internal force of the bolt in the field. As a result, many scholars have focused on observing the displacement of the excavated face to analyze the stability of the tunnel excavation. However, the excavation of the tunnel not only could destroy the displacement monitoring points, but also the excavation disturbance will cause large errors in the monitoring data.

In this project, strain gauges were glued on the fiberglass bolts to test the microstrain in the bolt. This method had been previously tried during field monitoring and shown to provide good results^21^. Monitoring the stress and strain field around the bolt would help to analyze the stability of the tunnel.

**Figure 14:**
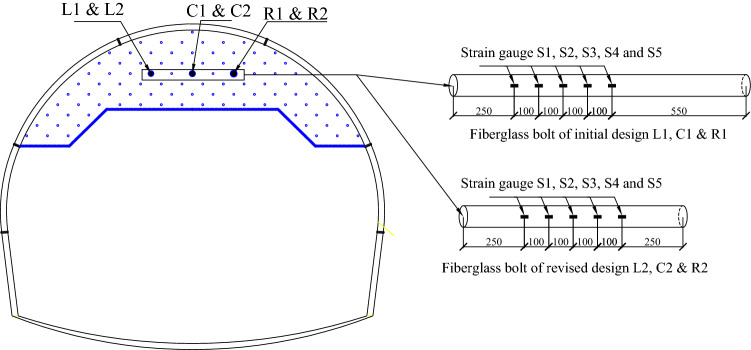
Location of the tested bolts in the excavation face and the strain gauge positions on the bolts.

Three instrumented fiberglass bolts were installed on the upper bench face each cycle, with five strain gauges arranged along each bolt and numbered S1, S2, S3, S4 and S5 from the outside to the inside in sequence. The position of the strain gauge along the bolts and the layout position of instrumented bolts on the excavation face are shown in Fig. 14.

**Figure 15:**
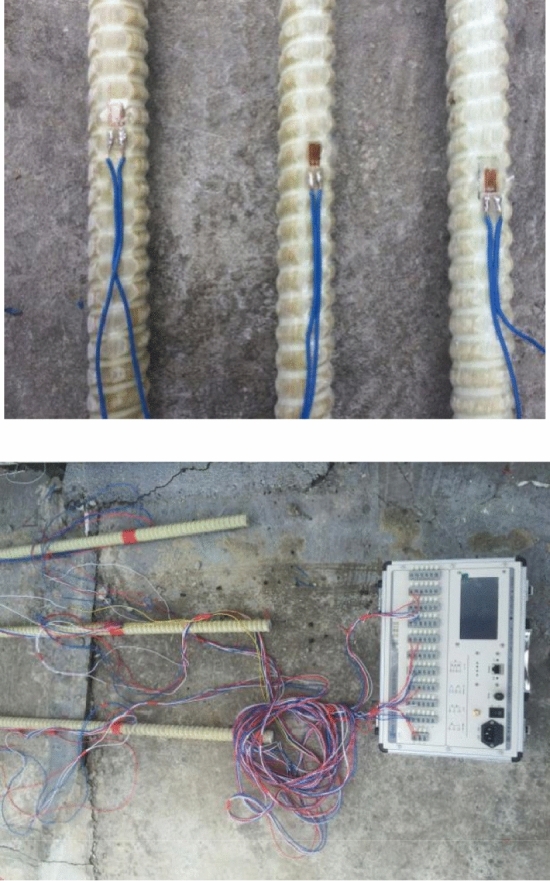
Attaching and initial testing of the strain gauges.

The strain gauges used a quarter bridge method with a common dummy gauge, and TST3822EN static resistance strain gauges were used to test the micro strain of the strain gauge. The strain gauge gluing details and the initial value testing of the micro strain are shown in Fig. 15. It should be noted that due to the fact that the bolt would be cut off during the excavation process, the wires were also easy to be cut off, with the different measuring points on the same bolt marked with wires of different colors. During the excavation process, care was taken not to damage the conductor.

When the face reinforcement construction started on the excavation face, the construction phase was following as: identifying the location, drilling the holes, putting the bolt in the hole and filling the hole with grout. It should be noted that the initial reading data was obtained after grouting the reinforcement. This was because the excavation face is in the process of slowly deforming and hence, the bolt installed first was subjected to a large force, and the bolts applied later were subjected to a relatively smaller axial force.

The installation of the monitoring point section was controlled according to the excavation advance distance of 1.2 m (spacing between two steel frames). After the excavation, the steel frames and shotcrete for the initial support were constructed immediately. Meanwhile, the numbered lines were matched to the corresponding channels for testing, data reading, and further analysis.

## Analysis and discussion of field monitoring data

As shown in Fig. 14 L1, C1 and R1 bolts were installed at section DK80 + 478. Strain gauges were installed at 0.25 m from the bolt head at 0.1 m intervals, with a total of 5 monitoring points. According to the 12 m long bolts proposed in the preliminary design, and the lap length was 4 m,the L2, C2, and R2 bolts would have been installed at section DK80 + 495. Based on the revised design, the bolt was 9 m long and the lap length was 3 m. The formula for determining the axial force in the bolt is shown in Eq. (1).

1 $$N = \varepsilon_{g} \cdot E_{g} \cdot A_{g}$$

*N*——Axial force of bolt(kN); $\varepsilon_{g}$——Strain value measured on site (µ ε); $E_{g}$ ——Elastic modulus of fiberglass bolt (GPa); $A_{g}$——Cross-sectional area of fiberglass bolt (m^2^).

**Figure 16:**
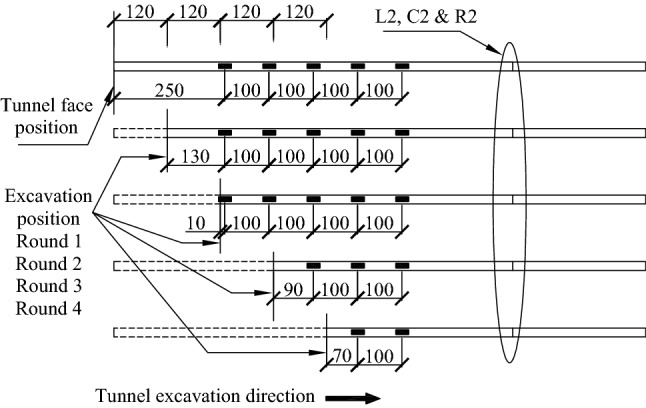
The relationship between the test points and excavation progress.

It should be noted that after each excavation advance, the axial force at the location of the face has been set to 0 and the excavation distance of 1.2 m, 2.4 m, 3.6 m, and 4.8 m have been defined as Round 1, Round 2, Round 3 and Round 4 respectively, as shown in Fig. 16.

**Figure 17:**
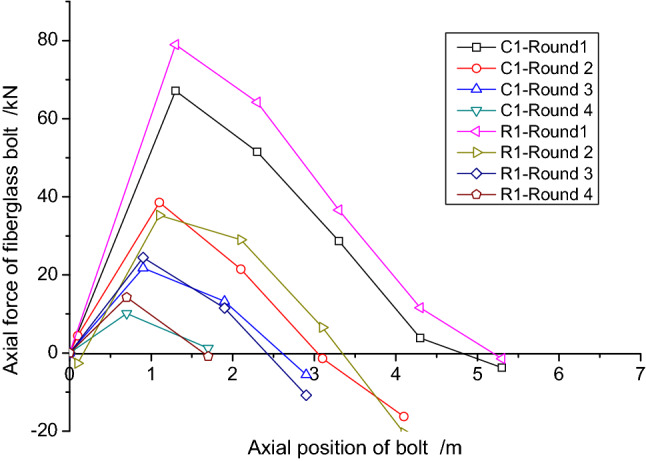
Axial force for C1 and R1 bolts.

**Figure 18:**
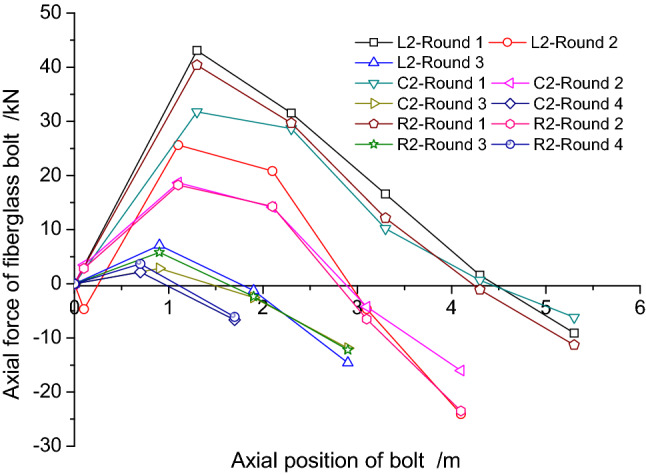
Axial force for L2, C2 and R2 bolts.

As the L1 bolt was damaged during the construction, only the monitoring data for C1, R1, L2, C2 and R2 bolts were obtained as shown in Figs. 17 and 18, respectively. In order to show the distribution of axial force more clearly, the data from Rounds 1 ~ 4 are all shown based on the section where the actual bolts were located.

The following conclusions can be obtained from the data in Figs. 17 and 18:

1. The axial force of the bolt reached the maximum value during the excavation of Round 1. With the gradual advance of the excavation, the axial force in the bolt decreased with time. When the excavation face reached Round 4, the overall axial force was already small;
2. Based on the data, the axial force values for the C1 and R1 bolts were significantly greater than the L2, C2 and R2 bolts. The reinforcement length of the bolt had an important impact on the stress state of the bolt;
3. The axial force value at the testing point gradually increased when it was closer to the excavation face;
4. The stress state of some of the testing points in S4 and S5 were in a compressive state, which was caused by not accounting for the initial reinforcement stress value. Meanwhile, the pressure value increased initially and then decreased, which showed a state of unloading initially and then loading.

**Figure 19:**
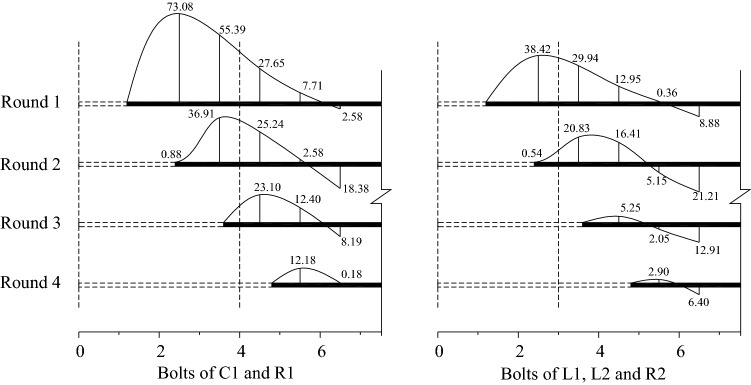
The axial force distribution in the bolts (The unit of axial force: kN).

Concerning the construction conditions, as shown in Fig. 19, the vertical dashed lines indicate the lap range of the bolts, and the values shown in the figure are the average values of all the axial forces in the bolts under the same conditions. The values at the upper part of the bolt indicate that the bolt was under tension and the values at the lower part indicate that the bolt was under compression. As can be seen from Fig. 19, the increase in axial force caused by excavation within the lap area was relatively large. After the excavation exceeded the lap area, the axial force of the bolt decreased dramatically, and the further away from the lap area, the smaller the value.

From Fig. 19, it can be seen that the axial force in the 12 m fiberglass bolt used as part of the initial design was larger than the value for the 9 m long bolt used in the revised design. In addition, by comparing the installation times for the two lengths of bolt in Table 5, the installation time of the bolt in the initial design was much longer than that in the revised design. During the period of the bolt installation, the surrounding rock of the excavated face that have been reinforced in the previous excavation cycle and not excavated yet (called the lap joint) was relatively stable, and it also has to bear the pressure from the unreinforced rock mass. Although the rock mass in front of the excavation face would be gradually reinforced during the installation of the bolts, the pressure still gradually increased with time. As the installation time of the bolts in the initial design was longer than that in the revised design, the load in the lap joint was higher than in the latter. The process of excavating the rock mass can be regarded as an unloading process, and the load borne by the excavated rock mass is transferred to the rock surrounding the excavation face and the rock in front of the excavation face. As the process of stress transfer is very fast, the surrounding rock mass in front of the excavation face will bear more load. Since the initial support application cannot keep pace with the stress transfer, the axial force in the bolt in the initial excavation method was noticeably higher than in the revised design, as shown in Fig. 19. With the continuation of the excavation, especially after the lap joint had been excavated, the axial force of the bolt was obviously reduced, which indicates that the newly reinforced rock mass outside the lap joint bears less load.

**Table 5:** Comparison of the construction time for the initial design and the revised design.

Project details	Initial design	Revised design
Number of bolts per cross-section	182	98
Time for construction of one-cycle of bolting	6 d	2.5 d
Time for excavation of one cycle and the associated advance length	3d/8 m	2d/6 m
Average tunneling speed	0.88 m/d	1.33 m/d

**Figure 20:**
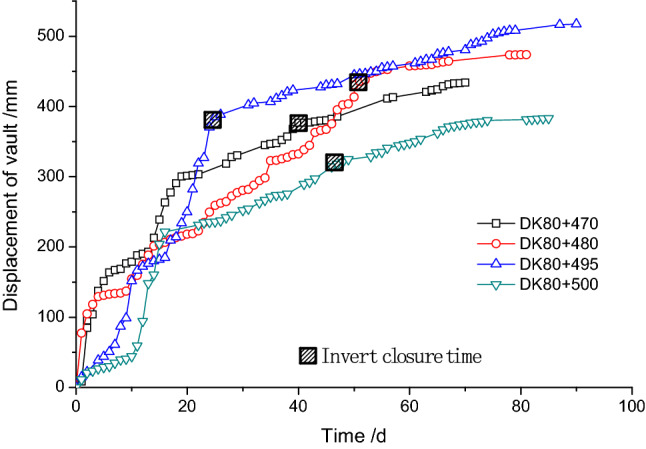
The excavation face vault settlement development curve under different face reinforcement.

Similarly, it can be seen from Fig. 20 that the deformation at sections DK80 + 470 and DK80 + 480 that were constructed using the initial design was significantly larger in the early stages of excavation than the constructed section using the revised design. This means that the load borne by the upper bench during the excavation in the initial design was significantly higher than that in the revised design. However, with the construction and excavation of the middle bench and the lower bench, it was found that the deformation in the initial design tends to settle, while the deformation in the revised design increases rapidly. This suggests that reducing the area of reinforced rock mass in the revised design affected the stability of the excavation of the lower bench. Based on how the settlement curve developed for the vault of the tunnel, as long as the inverted arch was closed, the overall deformation could be effectively controlled. In addition, the final settlement value all met the requirement of the allowed deformation limit (60 cm).

## Discussion

From the above analysis, due to the large number and long length of fiberglass bolts, the construction took a long time with the initial design, which led to the load within the lap joint increasing significantly. During the excavation process, the field stress transferred which resulted in a large deformation of the supporting structure in the early stages and large axial force in the bolts. However, in the revised design the bolt quantity was reduced resulting in a reduced construction time. The load borne by the lap joint in the revised design was small due to the small range of the lap joint. After the excavation, the axial force in the bolt was smaller, and the supporting deformation developed more slowly. However, the reduction in the area of reinforced rock mass had a negative effect on the deformation in the later period after the excavation.

Based on the analysis of the stress state in the bolts, the field deformation data and consideration of the reinforcement length, the choice of bolt length and lap length is impacted by two factors, i.e. the construction speed and the reinforcement strength. The higher the reinforcement strength, the longer the construction time, and the greater the load borne by the reinforced rock mass. During the excavation, the stress field will redistribute, and the internal force in the supporting structure in the upper bench will increase and the amount of deformation will be large. If the reinforcement area is reduced, the construction time will also be reduced, and the load borne by the reinforced rock mass will be smaller. On the other hand, the stability of the supporting structure of the middle and lower benches will be greatly affected by the reduced reinforcement. Therefore, finding the best balance between construction time and reinforcement strength is an important technical problem in the construction of fractured carbonaceous slate tunnels.

According to the results obtained from the research presented in this paper, the final value of the deformation can meet the requirements as long as the design can ensure the stability of the excavation face by reducing the exposure time of the excavated face, accelerating the excavation speed and closing the initial support ring early. In particular, the revised design in this project greatly improved the overall excavation progress as it could accelerate the construction time of the bolts.

## Conclusion

This paper described the issues related to tunnel stability during the construction of the Xingzishan tunnel as part of the Dalin railway in China. The tunnel was constructed through fractured carbonaceous slate and has a cross-section area of approximately 143.64 m^2^. In order to provide improved stability two methods of fiberglass face reinforcement design have been compared and analyzed. In addition, several reinforcement bolts within the top bench were instrumented with strain gauges to investigate the stress state in the reinforcement during the construction process. The following conclusions can be drawn from this study:

1. The reinforcement length of the bolt has an important impact on the stress state of the bolt.
2. The larger the lap joint of glass fiber bolts is, the longer the construction time is, and the greater the axial force of glass fiber bolts increases during excavation. After the lap joint has been excavated, the influence of the excavation on the stress change of the excavation face will gradually reduce.
3. The reduction in the extent of reinforced rock can affect the stability of the lower step excavation, but timely closure of the initial support can effectively control the overall deformation.
4. In similar tunnel projects with comparable ground conditions and dimensions, choosing grouted fiberglass bolts as an advanced reinforcement method with a lap length of 3 m can effectively control the face deformation.
5. The strain gauges measure the strain field only on a certain contact area, which is an inaccurate proxy for the overall mechanical behaviour of the bolt, and further research is still needed.

## Data Availability

Some or all data, models, or code that support the findings of this study are available from the corresponding author upon reasonable request; All data, models, and code generated or used during the study appear in the submitted article.
